# Cumulative Community-Level Lead Exposure and Pulse Pressure: The Normative Aging Study

**DOI:** 10.1289/ehp.10350

**Published:** 2007-09-06

**Authors:** Todd Perlstein, Jennifer Weuve, Joel Schwartz, David Sparrow, Robert Wright, Augusto Litonjua, Huiling Nie, Howard Hu

**Affiliations:** 1 Division of Cardiology, Department of Medicine, Brigham and Women’s Hospital, Harvard Medical School, Boston, Massachusetts, USA; 2 Department of Environmental Health, Harvard School of Public Health, Boston, Massachusetts, USA; 3 Veterans Affairs Boston Healthcare System, Boston, Massachusetts, USA; 4 Boston University Schools of Public Health and Medicine, Boston, Massachusetts, USA; 5 Channing Laboratory, Department of Medicine, Brigham and Women’s Hospital, Harvard Medical School, Boston, Massachusetts, USA; 6 Department of Emergency Medicine, Children’s Hospital, Harvard Medical School, Boston, Massachusetts, USA; 7 Department of Environmental Health Sciences, University of Michigan School of Public Health, Ann Arbor, Michigan, USA

**Keywords:** aging, epidemiology, human, lead exposure, pulse pressure

## Abstract

**Background:**

Pulse pressure increases with age in industrialized societies as a manifestation of arterial stiffening. Lead accumulates in the vasculature and is associated with vascular oxidative stress, which can promote functional and structural vascular disease.

**Objectives:**

We tested the hypothesis that cumulative community-level lead exposure, measured with K-X-ray fluorescence, is associated with pulse pressure in a cohort of adult men.

**Methods and results:**

In a cross-sectional analysis of 593 men not treated with antihypertensive medication, tibia lead was positively associated with pulse pressure (*p* < 0.001). Adjusting for age, race, diabetes, family history of hypertension, education, waist circumference, alcohol intake, smoking history, height, heart rate, fasting glucose, and total cholesterol-to-HDL ratio, increasing quintiles of tibia lead remained associated with increased pulse pressure (*p*_trend_ = 0.02). Men with tibia lead above the median (19.0 μg/g) had, on average, a 4.2-mmHg (95% confidence interval, 1.9–6.5) higher pulse pressure than men with tibia lead level below the median. In contrast, blood lead level was not associated with pulse pressure.

**Conclusions:**

These data indicate that lead exposure may contribute to the observed increase in pulse pressure that occurs with aging in industrialized societies. Lead accumulation may contribute to arterial aging, perhaps providing mechanistic insight into the observed association of low-level lead exposure with cardiovascular mortality.

In industrialized societies, pulse pressure (systolic minus diastolic blood pressure) increases with age, a trend that accelerates in the sixth decade when the diastolic blood pressure begins to decrease (Franklin et al. 1997). The increase in pulse pressure reflects arterial aging and progressive vascular stiffening (Lakatta and Levy 2003), with the predominant contribution from increased aortic stiffness (Mitchell et al. 2004). Vascular oxidative stress contributes to arterial aging (Lakatta 2003). Accordingly, known contributors to vascular oxidative stress including obesity (Kwagyan et al. 2005), smoking (Mahmud and Feely 2003), hyperglycemia (van Dijk et al. 2002), and dyslipidemia (Miyagi et al. 2002) are associated with increased pulse pressure.

Lead exposure is also associated with vascular oxidative stress (Vaziri 2002). *In vivo* (Vaziri et al. 1999) and *in vitro* (Vaziri and Ding 2001) studies of lead demonstrate increased vascular reactive oxygen species generation. Lead accumulates in the vasculature of the lead-exposed rat and remains after the exposure has ended (Malvezzi et al. 2001). The lead-exposed rat develops hypertension ameliorable by antioxidant therapy (Vaziri et al. 1997). These findings suggest that accumulation of lead in the arterial tree may contribute to arterial stiffness by inducing oxidative stress.

In industrialized societies, accumulation of bone lead is many times greater than that observed in cultures that do not use lead (Drasch 1982). Therefore, bone lead may serve as a proxy marker of lead accumulated in the arterial tree. In fact, human autopsy studies demonstrate age- and dose-dependent aortic lead deposition and suggest that the aorta is the next most lead-avid tissue after bone (Barry and Mossman 1970; Schroeder and Tipton 1968). Although public health initiatives have been successful at lessening environmental lead exposures in the United States (Muntner et al. 2005), low-level lead exposure remains an important contributor to all-cause and cardiovascular mortality (Menke et al. 2006).

The effect of low-level environmental exposure to lead on blood pressure is an area of ongoing scientific debate. Some investigators have found the relationship between low-level lead exposure and blood pressure to be inconsistent and weak (Nawrot et al. 2002; Staessen et al. 1994), but several toxicologic studies by have found that lead elevates blood pressure (Khalil-Manesh et al. 1993; Vaziri et al. 1997; Victery et al. 1982). Other investigators have noted the consistency of the effect size of the blood lead–blood pressure association, and its significance in meta-analyses (Navas-Acien et al. 2007; Schwartz 1991, 1995). A limitation of this body of work is the use of lead in blood as a metric of exposure, where the median residence time of lead is measured in days. Yet autopsy studies indicate that around 95% of lead in the adult human body is deposited in the skeleton, and to the extent that the lead’s effect on blood pressure can be attributed to chronic exposures, a longer averaging time for exposure would be more relevant for evaluating these effects. For example, using K-X-ray fluorescence (KXRF) to directly measure levels of lead retained in bone, we have found that bone lead, compared with blood lead, more accurately reflects cumulative lead exposure (Hu et al. 1996b). We have also found bone lead level to be more strongly associated than blood lead level with blood pressure and hypertension in adult men (Cheng et al. 2001; Hu et al. 1996a).

We examined the cross-sectional association of community-level lead exposure with pulse pressure in the Normative Aging Study, a longitudinal cohort of men. We analyzed this association using both bone and blood lead levels, anticipating that the former, a more accurate indicator of cumulative lead exposure, would be more strongly associated with pulse pressure.

## Methods

The Human Subjects Committees of the Boston VA Medical Center, the Brigham and Women’s Hospital, and the Harvard School of Public Health approved this research.

### Study population

Participants were from the Normative Aging Study (NAS), a longitudinal study of aging established by the Veterans Administration in 1961. Male volunteers from the Greater Boston, Massachusetts, area were screened at entry and enrolled in the study if they had no history of heart disease, hypertension, diabetes, cancer, peptic ulcer, gout, recurrent asthma, bronchitis, or sinusitis. Those with either a systolic blood pressure > 140 mmHg or a diastolic blood pressure > 90 mmHg were disqualified. Between 1963 and 1968, 2,280 men were enrolled; their ages at entry ranged from 21 to 80 years. Participants were asked to return for follow-up examinations every 3–5 years, and the attrition rate was roughly 1% per year over the life of the study. Beginning in 1991, we invited the men still being monitored by the NAS to take part in a study of lead exposure, as assessed by KXRF measurements. The study was approved by the human subjects committees of both the Boston Veterans Administration Medical Center and the Brigham and Women’s Hospital.

### Bone and blood lead measurements

NAS participants who gave their informed consent reported to the outpatient General Clinical Research Center of the Brigham and Women’s Hospital in Boston, where we measured their bone lead levels at two sites (the midtibial shaft and the patella) with a KXRF instrument (ABIOMED, Inc., Danvers, MA). The physical principles, technical specifications, and validation of this instrument have been described in detail elsewhere (Burger et al. 1990; Hu et al. 1990a, 1990b). Because the instrument provides a continuous unbiased point estimate (micrograms of lead per gram of bone mineral) that oscillates around the true bone lead value, it sometimes produces negative point estimates when the true bone lead value is close to zero. The instrument also provides an estimate of the uncertainty associated with each measurement that is derived from a goodness-of-fit calculation of the spectrum curves and is equivalent to a standard deviation (SD) if multiple measurements were taken. Although a minimally detectable limit calculation of twice this SD has been proposed for interpreting an individual’s bone lead estimate (Gordon et al. 1993), retention of all point estimates has been shown to make better use of the data in epidemiologic studies (Kim et al. 1995). The technicians measuring bone lead were blinded to the participants’ health status. Thirty-minute measurements were taken at the midshaft of the left tibia and at the left patella. *A priori* we chose to examine tibia lead as a marker of cumulative lead exposure because tibia bone is mostly cortical bone, whereas the patella is mostly trabecular bone and has a greater turnover rate (Hu et al. 1996b).

Blood samples were obtained and analyzed by graphite furnace atomic absorption spectroscopy (GF-AAS; ESA Laboratories, Chelmsford, MA); this instrument was calibrated after every 21 samples with National Bureau of Standards’ blood lead standards materials (Gaithersburg, MD). The limit of detection for the GF-AAS method is < 1 μg/dL; thus, values are expressed as integers going down to 0 μg/dL. Ten percent of the samples were run in duplicate; at least 10% of the analyses were controls, and 10% were blanks. In tests on reference samples from the Centers for Disease Control and Prevention (Atlanta, GA), the precision (the coefficient of variation) ranged from 8% for concentrations between 10 and 30 μg/dL to 1% for higher concentrations. In comparison with a National Bureau of Standards’ target of 5.7 μg/dL, 24 measurements by this method gave a mean of 5.3 μg/dL with an SD of 1.23 μg/dL.

### Blood pressure measurements

During each clinical visit, a physician using a standard mercury sphygmomanometer with a 14-cm cuff measured the participant’s blood pressure. With the participant seated, the systolic blood pressure and fifth-phase diastolic blood pressure were measured once in each arm to the nearest 2 mmHg. For this study, the mean of the right and left arm measurements was used as each participant’s systolic and diastolic blood pressures. Pulse pressure was calculated as the mean systolic minus the mean diastolic blood pressure. Heart rate was recorded as beats per minute.

### Physical parameters and medical history

For each clinical visit, the NAS participant reported to the study center in the morning after an overnight fast and abstinence from smoking. At the start of the visit, height and weight were measured. Thereafter, a physician took a complete medical history and confirmed the identity and purpose of medications taken daily. Medications were considered anti-hypertensive if they included a beta-blocker, calcium channel blocker, diuretic, or other vascular agent prescribed by the participant’s physician. The participant also indicated whether his mother or father had hypertension that was diagnosed by a physician. Alcohol and dietary intake were assessed with a standardized semiquantitative food frequency questionnaire (Willett et al. 1988), in which participants reported the average frequency of each listed food item consumed in the previous year. In the present study, we examined sodium and calcium intakes, which we calculated by multiplying the frequency of intake by the nutrient content of the food items. We solicited information on current and past history of smoking using questions developed for the American Thoracic Society (Ferris 1978).

### Statistical analysis

The present analysis is a cross-sectional examination of the association of blood and bone lead levels with pulse pressure in subjects with these measurement made in the years 1991–1997. The participants for the present study were a subgroup of the NAS cohort who underwent at least one KXRF bone lead measurement and were not on antihypertensive therapy at the time of this measurement. Of the 1,262 men who were seen for their regularly scheduled visits between August 1991 and December 1997, 840 (66.6%) underwent KXRF measurement. The most common reason given for not having a measurement was the inconvenience involved in making another visit to the bone lead laboratory on a separate day. As a standard quality-control procedure, we excluded seven men who had high uncertainty estimates (> 10 μg/g) for bone lead measurement (Hu et al. 1996b). A comparison of the group of men who had KXRF examinations with the group of men who either did not have KXRF examinations or whose bone lead measurements had a high degree of uncertainty revealed no significant differences with respect to age, race, body mass index (BMI), smoking, alcohol consumption, systolic or diastolic blood pressure, family history of hypertension, dietary sodium or calcium intake, and blood lead level (Cheng et al. 2001; Hu et al. 1996a). Among the 833 participants in the bone lead study, 233 were on antihypertensive therapy at the time of their study visit, and seven men did not have blood pressure data to calculate pulse pressure. We therefore included 593 men in this analysis. The blood pressure measurement typically preceded the lead determinations, and the median (interquartile range) number of days between these measurements was 18 (8–39).

For each of the two lead biomarkers (blood lead and tibia lead), we used multiple linear regression to compare the mean pulse pressure across quintiles of the lead biomarker. We used quintiles of lead level to limit the influence of outliers and to not assume a linear relationship between lead level and pulse pressure. We determined covariates for our core models based on known determinants of bone and blood lead level, blood pressure, risk factors for arterial aging, and physiologic determinants of pulse pressure. Our regression analyses were all adjusted for the following variables, all of which were assessed at the time of bone lead measurement: age (years), age squared, height (meters), race (white vs. nonwhite), heart rate (beats/minute), waist circumference (centimeters), diabetes, family history of hypertension (yes/no), education level achieved, smoking (pack-years), alcohol intake (grams per day), fasting plasma glucose (mmol), and ratio of total cholesterol to HDL (high-density lipoprotein) cholesterol (Hu et al. 1996a, 1996b; Kwagyan et al. 2005; Mahmud and Feely 2003; Miyagi et al. 2002; van Dijk et al. 2002). We did not adjust our analyses for systolic and diastolic blood pressure, from which the pulse pressure is mathematically derived. We used an *F*-test (with *p* = *Pr*[*F_5-1,593-5_* *> F* ]) to evaluate the overall association between level of lead biomarker and pulse pressure, and we computed tests of linear trend by fitting models with an ordinal term, which took on the values of each biomarker quintile (i.e., 1, 2, 3, 4, 5). We fit additional models that further adjusted for dietary sodium and calcium as well as total caloric intake.

All analyses were conducted with the SAS software program (version 8.2; SAS Institute Inc., Cary, NC) with *p* < 0.05 as the level of statistical significance. All authors had full access to the data and take responsibility for its integrity. All authors have read and agree to the manuscript as written.

## Results

The blood lead levels of the study population ranged from < 1 to 35 μg/dL, with a mean (± SD) of 6.12 ± 4.03 μg/dL. These values are representative of community-level (i.e., nonoccupational) exposure in the U.S. general population in this age range (Pirkle et al. 1994). The mean levels (± SD) of blood lead among the first through fifth quintiles were 2.3 ± 0.8, 3.9 ± 0.3, 5.4 ± 0.5, 7.4 ± 0.6, and 12.4 ± 4.4 μg/dL, respectively. The mean levels of tibia lead among the first through the fifth quintiles were 7.4 ± 3.2, 14.1 ± 1.4, 18.9 ± 1.4, 24.9 ± 2.2, and 40.9 ± 14.0 μg/g bone, respectively.

We examined participants’ characteristics in relation to quintile of tibia lead level to provide insight into potential confounders of the association between lead and pulse pressure (Table 1). Of particular note, higher tibia lead level was associated with age, smoking, lower education level, and shorter height. None of these characteristics varied across quintile of blood lead level (data not shown) [see Hu et al. (1996b) for more details on determinants of bone and blood lead levels].

**Figure 1:**
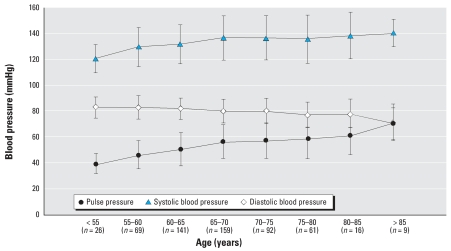
Arterial pressure parameters (mean ± SD) by age group.

We examined the progression of systolic and diastolic blood pressure and pulse pressure with age, categorized in 5-year increments (Figure 1). As demonstrated in other industrialized populations, the systolic blood pressure and pulse pressure increased with age, whereas the diastolic blood pressure decreased after the sixth decade (Franklin et al. 1997; Lakatta and Levy 2003).

We evaluated unadjusted, age-adjusted, and multivariable-adjusted correlations of blood and bone lead levels with systolic and diastolic blood pressure (Table 2). Blood lead level tended to be directly associated with systolic and diastolic blood pressure. Tibia lead level tended to be directly associated with systolic blood pressure and tended to be inversely associated diastolic blood pressure. After multivariable adjustment, the direct association of blood lead with diastolic blood pressure remained significant.

We observed significant differences in pulse pressure across quintiles of bone lead but not blood lead after multivariable adjustment. Tibia lead–level quintile was significantly associated with pulse pressure (overall *F*-test, *p* < 0.01), with pulse pressure increasing with tibia lead–level quintile (*p*_trend_ = 0.02; Table 3). Men with tibia lead level above the median (19.0 μg/gm) had, on average, a mean pulse pressure that was 4.2 mmHg greater [95% confidence interval (CI), 1.9–6.5] than men with tibia lead levels below the median. Blood lead was not associated with pulse pressure (overall *F*-test, *p* = 0.20). Dietary intake of sodium, dietary intake of calcium, and hematocrit were all added individually to our models but did not substantially change these results. In addition, we did not find a significant interaction between dietary calcium intake and tibia lead quintile in determining the pulse pressure. The association of patella lead with pulse pressure was of borderline statistical significance (data not shown).

Analyses were repeated excluding the 14 nonwhite participants; the association of tibia lead with pulse pressure was not changed. We performed additional analysis including men treated with antihypertensive therapy (total *n* = 826), and adjusting for antihypertensive therapy (using vs. not using). In this analysis, tibia lead level remained associated with pulse pressure (Overall *F*-test *p* = 0.02, *p*_trend_ = 0.04), though the effect of tibia lead quintile on pulse pressure was somewhat attenuated [highest vs. lowest quintile, difference in pulse pressure = 1.9 mmHg (95% CI, 1.4–5.1)].

## Discussion

In this study of 593 older men, cumulative community-level lead exposure was independently associated with increased pulse pressure. Specifically, we observed this association with respect to bone lead level, but not blood lead level. These results are consistent with the concept that bone lead level is a better indicator of cumulative lead exposure. These data may provide some mechanistic insight into the association of low-level lead exposure with cardiovascular mortality (Menke et al. 2006).

The pulse pressure—the difference between the systolic and the diastolic blood pressures—is receiving increasing attention as both an indicator of and a risk factor for cardiovascular disease (Safar et al. 2003). Our data support the well-described progression of pulse pressure with age, with progressively higher systolic pressures throughout adulthood, and the diastolic pressure leveling off among men in the sixth decade and declining in older men (Franklin et al. 1997).

The progression of pulse pressure with age is largely attributed to progressive aortic stiffening (Mitchell et al. 2004). Age-related arterial stiffening is attributable to structural and functional changes in the vasculature (Lakatta and Levy 2003). Structurally, vascular aging is associated with increased collagen content, increased elastin fractures, increased calcification, and reduced elastin content. Functionally, vascular aging is associated with impaired endothelial function and endothelium-dependent vasodilation (Gerhard et al. 1996). A fundamental and shared mechanism for vascular structural and functional changes is vascular oxidative stress (Lakatta 2003; Touyz 2004). Observations in lead-exposed animals strongly suggest that vascular oxidative stress plays a key role in the pathophysiology of lead-induced hypertension and vascular disease (Farmand et al. 2005; Malvezzi et al. 2001; Ni et al. 2004; Vaziri 2002; Vaziri and Ding 2001; Vaziri and Sica 2004; Vaziri et al. 1997, 1999a, 1999b, 2001, 2003). Human studies indicate that lead accumulates in the aorta (Barry and Mossman 1970; Schroeder and Tipton 1968) and that lead exposure is associated with oxidative stress in humans (Gurer-Orhan et al. 2004; Ye et al. 1999), although to our knowledge the association of lead exposure with vascular oxidative stress in humans has not been specifically examined.

In this study, tibia bone lead was more strongly associated with pulse pressure than was patella bone lead (data not shown). The tibia bone is mostly cortical bone, whereas the patella is mostly trabecular bone and has a greater turnover rate (Hu et al. 1996b). Tibia lead has a longer half-life and is considered a better marker of cumulative lead exposure than patella lead. The stronger association of tibia lead with pulse pressure is consistent with an effect of long-term lead exposure on vascular structure and function. Interestingly, blood lead level was positively associated with diastolic blood pressure, causing us to speculate that circulating lead may have more of an influence on vascular tone than vascular structure, though this requires further exploration.

We found that men with bone lead levels above the median had pulse pressures that were on average 4.2 mmHg higher compared with men with lower bone lead levels. The magnitude of this association is numerically small but potentially clinically significant. In the Conduit Artery Function Evaluation (CAFE) study of the Anglo-Scandinavian Cardiac Outcomes Trial (ASCOT) comparing amlodipine ± perindopril versus atenolol ± thiazide in the treatment of hypertension, a 3-mmHg decrement in central pulse pressure conferred a cardiorenal advantage to the amlodipine ± perindopril strategy (Williams et al. 2006). Pulse pressure is an important indicator of cardiovascular risk becasue pulse pressure is associated with inflammation (Abramson et al. 2002), coronary heart disease (Franklin et al. 1999), and cardiovascular death (Domanski et al. 2001). The association we have demonstrated between lead exposure and pulse pressure may provide mechanistic insight into the association of lead exposure with cardiovascular death (Menke et al. 2006).

Because this is a cross-sectional study, we cannot explicitly examine the temporal association between lead exposure and subsequent pulse pressure or individual changes in pulse pressure. Nonetheless, our bone lead measures, particularly tibia lead, do reflect exposures that occurred over the past years to decades, suggesting a temporal ordering of the association that we observed between bone lead and pulse pressure. Moreover, reverse causation—the situation in which pulse pressure affects lead exposure—is highly unlikely. Blood pressure was measured once in each arm; averaging these values increases the precision of our measurement. It is possible, however, that differences in the blood pressure between the arms due to subclavian stenosis or other processes may introduce error into our blood pressure determination and limit the precision of our lead effect estimates. Although our results were adjusted for numerous important potential confounders, we cannot rule out the possibility that the association we found derives from confounding by unmeasured factors or that categorical tibia lead level may not entirely account for the association of bone lead with aging. The data suggest that there may be a threshold to the effect of lead exposure on pulse pressure, because only the top two quintiles of tibia lead were associated with a mean pulse pressure greater than that of the first quintile. It is possible that selective survival biased our findings in this older cohort of men, in that survival may be related to lower lead exposures and lower pulse pressure; even so, such bias would tend to make our results a conservative estimate of the adverse association between bone lead and pulse pressure.

Although lead exposure has been associated with elevated blood pressure in women (Nash et al. 2003) and blacks (Vupputuri et al. 2003), our findings are limited to Caucasian men. This cohort of men had exposures to lead before major public health reforms that removed lead from paint and gasoline, and they may have been further exposed in their military careers, making their typical historical exposures higher than current exposure levels. A sufficient threshold of arterial aging needs to be achieved before brachial pulse pressure begins to widen; therefore, the association of lead and pulse pressure may not be present in younger populations (O’Rourke and Hashimoto 2007). Also, in older populations, the association of lead exposure with pulse pressure may be attenuated by selective survival, because both lead exposure and pulse pressure are strong predictors of mortality (Domanski et al. 2001; Schober et al. 2006). Finally, studies directly examining the association of lead exposure with vascular function and pulse hemodynamics are necessary to better define the relationship between lead exposure and arterial aging.

In conclusion, we found that cumulative lead exposure, as reflected by bone lead level, was independently associated with increased pulse pressure in a cohort of middle-aged and older men with community-level lead exposure. These findings implicate vascular accumulation of lead in the pathogenesis of vascular stiffening, a mechanism consistent with the findings of aortic lead deposition in humans and increased vascular oxidative stress in the lead-exposed animal. Future work will determine the effect of lead exposure on the progression of pulse pressure with aging. We suggest that further examinations of the association of lead exposure on arterial pressure should use bone lead level as an indicator of cumulative lead exposure.

## Figures and Tables

**Table 1:** Characteristics of men by quintile of tibia lead (μg/g).

	Quintile tibia lead level [mean (range)]					
	Lowest (*n* = 111)	2 (*n* = 119)	3 (*n* = 122)	4 (*n* = 119)	Highest (*n* = 122)	
Characteristic	7.4 (−3 to 11)	14.1 (12 to 16)	18.9 (17 to 21)	24.9 (22 to 29)	40.9 (30 to 126)	*p*-Value
Age (years)	63.0 ± 7.0	64.4 ± 6.9	66.8 ± 7.4	68 ± 7.3	70.5 ± 6.7	< 0.001
Race (nonwhite)	2 (1.8)	2 (1.7)	2 (1.6)	3 (2.5)	5 (4.1)	0.21
Diabetes mellitus diagnosis	4 (3.6)	4 (3.4)	7 (5.7)	6 (5.0)	13 (11)	0.02
Smoking history (pack-years)	13 ± 14	14 ± 15	18 ± 17	20 ± 16	19 ± 17	0.002
Alcohol history (g/day)	12 ± 15	12 ± 16	15 ± 21	16 ± 19	11 ± 15	0.22
Dietary sodium intake (mg/day)	3,559 ± 1,562	3,930 ± 1,916	3,925 ± 1,736	3,850 ± 1,723	4,141 ± 2,055	0.21
Dietary calcium intake (mg/day)	819 ± 393	897 ± 492	856 ± 453	847 ± 524	833 ± 444	0.77
Family history of hypertension	17 (18)	37 (34)	30 (27)	24 (22)	16 (14)	0.11
Education level (graduated high school)	71 (64)	71 (60)	59 (48)	58 (49)	42 (34)	< 0.001
Systolic blood pressure (mmHg)	133 ± 16	129 ± 14	133 ± 15	137 ± 19	137 ± 17	0.001
Diastolic blood pressure (mmHg)	83 ± 9.0	81 ± 7.9	80 ± 9.7	80 ± 10	80 ± 9.3	0.008
Pulse pressure (mmHg)	50 ± 14	49 ± 12	53 ± 14	57 ± 15	58 ± 14	< 0.001
Heart rate (beats/min)	75 ± 10	72 ± 10.3	72 ± 9.6	73 ± 11	74 ± 12	0.09
BMI (kg/m^2^)	28 ± 3.6	27 ± 3.5	28 ± 3.9	28 ± 3.6	28 ± 3.4	0.92
Waist circumference (cm)	98 ± 10	97 ± 9.9	98 ± 9.8	98 ± 9.7	98 ± 8.9	0.85
Height (m)	1.75 ± 0.07	1.74 ± 0.06	1.74 ± 0.06	1.72 ± 0.06	1.71 ± 0.06	< 0.001
Fasting plasma glucose (mmol)	5.83 ± 1.1	5.78 ± 1.3	6.00 ± 2.2	6.05 ± 1.4	6.11 ± 1.6	0.41
Hematocrit (%)	44 ± 2.8	44 ± 2.9	44 ± 2.7	44 ± 3.0	44 ± 3.5	0.40
Total cholesterol to HDL ratio	5.1 ± 1.5	5.0 ± 1.4	4.9 ± 1.4	4.8 ± 1.2	5.1 ± 1.5	0.54
Blood lead level (μg/dL)	4.9 ± 2.9	5.3 ± 3.6	5.6 ± 3.5	6.7 ± 3.8	8.1 ± 5.2	< 0.001

Comparisons are by overall *F*-test for continuous variables and by chi-square test for categorical variables. Values are mean ± SD or no. (%).

**Table 2:** Spearman correlations between lead bio-marker levels and blood pressure (BP) components.

	Systolic BP		Diastolic BP	
	*r*	*p*-Value	*r*	*p*-Value
Tibia lead level (μg/g)				
Unadjusted	0.13	< 0.01	−0.14	< 0.01
Age-adjusted	0.05	0.21	−0.06	0.14
Multivariable-adjusted	0.06	0.15	−0.02	0.63
Blood lead level (μg/dL)				
Unadjusted	0.08	0.05	0.09	0.03
Age-adjusted	0.07	0.11	0.11	0.01
Multivariable-adjusted	0.05	0.28	0.12	0.01

**Table 3:** Multivariable-adjusted group mean differences in pulse pressure by tibia lead and blood lead level quintile.

Quintile	Adjusted mean difference in pulse pressure	95% CI	*P*_trend_
Tibia lead			0.02
Highest	2.58	−1.15 to 6.33	
Fourth	2.64	−0.93 to 6.21	
Third	−0.73	−4.27 to 2.82	
Second	−3.02	−6.48 to 0.44	
Lowest	0.00	Referent	
Blood lead			0.82
Highest	−1.49	−4.93 to 1.94	
Fourth	−1.39	−4.94 to 2.15	
Third	−2.56	−5.78 to 0.67	
Second	−4.37	−7.88 to −0.86	
Lowest	0.00	Referent	

Analyses are adjusted for age (years), age squared, height (m), race (white vs. nonwhite), heart rate (beats/min), education level achieved, waist circumference (cm), diabetes, family history of hypertension (yes/no), smoking (pack-years), alcohol intake (g/day), fasting plasma glucose (mmol/L), and ratio of total cholesterol to HDL cholesterol.
